# Automatic generation of gene finders for eukaryotic species

**DOI:** 10.1186/1471-2105-7-263

**Published:** 2006-05-21

**Authors:** Kasper Munch, Anders Krogh

**Affiliations:** 1 Bioinformatics Centre, University of Copenhagen, Universitetsparken 15, 2100 Copenhagen Ø, Denmark

## Abstract

**Background:**

The number of sequenced eukaryotic genomes is rapidly increasing. This means that over time it will be hard to keep supplying customised gene finders for each genome. This calls for procedures to automatically generate species-specific gene finders and to re-train them as the quantity and quality of reliable gene annotation grows.

**Results:**

We present a procedure, Agene, that automatically generates a species-specific gene predictor from a set of reliable mRNA sequences and a genome. We apply a Hidden Markov model (HMM) that implements explicit length distribution modelling for all gene structure blocks using acyclic discrete phase type distributions. The state structure of the each HMM is generated dynamically from an array of sub-models to include only gene features represented in the training set.

**Conclusion:**

Acyclic discrete phase type distributions are well suited to model sequence length distributions. The performance of each individual gene predictor on each individual genome is comparable to the best of the manually optimised species-specific gene finders. It is shown that species-specific gene finders are superior to gene finders trained on other species.

## Background

Hidden Markov models (HMMs) have been extensively used for modelling genes. *Ab initio *HMM gene finders for eukaryotes include Genscan [1], Augustus [2], HMMgene [3,4], GeneMark.HMM-E [5], Genie [6], TigrScan and GlimmerHMM [7], Unveil and Exonomy [8], SNAP [9], and others. Examples of non-HMM approaches are GeneID [10,11], GlimmerM [12], and MZEF [13]. GenelD applies Markov models to score sequence content and signal in a hierarchical manner. GlimmerM uses decision trees and Interpolated Markov Models. MZEF uses quadratic discriminant analysis to predict internal exons. These predictors all use a single genomic sequence. Examples of approaches using two genomic sequences are SLAM [14], SGP-2 [15], TWINSCAN [16], and DoubleScan [17]. These use homology information in alignments that improves prediction accuracy relative to single genome predictors. EHMM [18], Phylo-HMM [19], and N-Scan [20] use more than two genomic sequences, taking advantage of the fact that the molecular evolution of a sequence position is governed by its function. Gene finders using multiple genomes have a higher accuracy but training sets with species of an appropriate evolutionary distance may be hard to come by. Single-genome approaches have the advantage that they do not require complex training material and can predict non-conserved genes.

*Ab initio *gene finders are trained and customised for one or a few organisms and may perform well on other organisms that share characteristics such as sequence signals, length distributions of gene elements, and overall sequence composition. These gene characteristics, however, vary substantially between species. Establishing to what extent this is the case requires extensive knowledge of gene structure that is not available in the early stages of genome exploration.

The creation of a gene finder for a novel genome includes two laborious and non-trivial tasks. First a training set of reliable gene structures must be generated. Secondly, a gene model must be built, customised, and trained. TigrScan, SNAP, GlimmerHMM, GlimmerM, Unveil, and Exonomy include tools to ease the task of re-training model parameters. For TigrScan this involves some expertise because individual parts of the model must be trained separately. SNAP, GlimmerHMM, GlimmerM, Unveil, and Exonomy have automated much of this task, but creating a training set is still left to the user. In addition, none of the existing methods allow for easy adjustment of the gene model. This is necessary in cases where the available training material only supports a less detailed model.

The work involved in obtaining a training set as well as the limited modelling expertise of most end users are in our view the main obstacles to producing gene predictors for novel genomes. As a result, genome projects often do not have species-specialised gene finders. Apart from a novel approach to length modeling of gene features, the approach presented here is unique in the respect that it is the only gene prediction package that fully integrates the task of creating a training set, as well as adjusting the detail of the model to the quality of the training material. This allows a non-expert to produce a gene finder for a novel genome directly from a set of mRNAs from the same organism. Our implementation, Agene, thus fully automates the task of building and training a species-specialised gene finder from mRNA evidence. For the majority of genomes the resulting gene finders do as well or better than manually tuned gene finders. Automatic gene annotation has already been implemented for prokaryotic organisms. These methods include Easygene [21], Glimmer [22,23], and versions of GeneMark [24-26].

## Methods

An HMM consists of a hidden layer of states that emit observable events. In gene finding, the states in the hidden Markov chain correspond to intergenic regions and gene structure elements, e.g. coding regions and introns. Each state emits nucleotides that constitute the DNA sequence in corresponding regions. The emissions of bases may be conditional on the occurrence of neighbouring bases within the sequence. This enables the HMM to model higher order dependencies of base frequencies. A second order model will thus model the frequencies of base triplets in the sequence.

When applying an HMM to find genes, the task is to find the most likely annotation of the sequence given the model. This process is called decoding. Estimation of the model parameters from known gene structures is referred to as training. Here the parameters are optimised in an iterative procedure. In each iteration the sequences of the training set are decoded and all parameters are then assigned the maximum likelihood estimates given the sequences. This process is stopped when the change in overall likelihood of the model becomes sufficiently small. The theoretical aspects of HMMs are well covered elsewhere [27].

### Gene model

**Figure 1:**
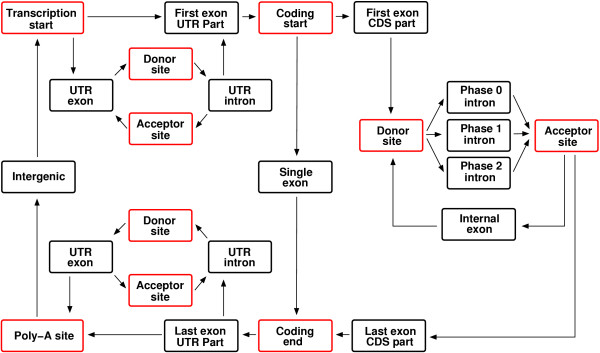
**Overview of gene model**. Each box represents a separately modelled gene structure element. Red boxes are weight array matrices. Black boxes are length modelled elements. For clarity the intron models that assures that splicing does not introduce stop codons are not shown.

Agene generates a suitable gene model by automatically customising HMM state structure to fit the information supplied by the training set. Firstly, a gene model is assembled by dynamically combining an array of different sub-models. The automation of this task means that only gene elements generally featured by eukaryotes are considered. The model shown in Figure 1 is an outline of the most detailed gene model generated by Agene. Each block shown represents a sub-model of a gene feature. The sub-models for initial, internal, last, and single coding exons are obligatory whereas the sub-models for UTR elements are not. In many cases the available training set does not support all feature sub-models. UTRs are often not reliably annotated and in these cases the gene model is dynamically adjusted so that UTR exons and UTR introns are modelled as part of the intergenic region. In cases where only the coding regions are annotated the UTR-parts of first and last exons are treated this way as well.

Secondly, sub-models of variable length gene features include a length distribution of the modelled gene feature. This length distribution is implemented as state structure customised for each feature (see below). In some cases one or more of the obligatory sub-models are not sufficiently represented in the training set to fit separate length distributions. To remedy this situation Agene fits a shared length distribution for one or more types of exons by pooling length statistics. A shared distribution for internal and single exons are fitted if either of these are insufficient in number. This will happen in cases where single exon genes are rare or when the majority of genes have at most two exons. If all types of exons are insufficient in number, as when the training set is small, a shared distribution is fitted for all types of exons. In cases where only a small fraction of genes have more than one exon the number of introns may be insufficient to fit a detailed distribution. In this case Agene uses a less detailed length distribution with only four phases (see below). In order to be able to detect overlapping genes on the opposite strand, genes are modelled one strand at a time.

#### Content modelling

The coding regions are modelled as inhomogeneous Markov models with a three-periodicity and fourth order emissions to capture information in codon statistics of the three reading frames. The intergenic model and the intron models share fourth order emission probabilities. Not doing this will make the algorithm tend to predict genes around regions in intergenic sequence with a sequence composition similar to that of introns. This is not a severe compromise assuming that the amount of gene related information in deep intron sequence is small. Eight intron models that share both transition and emission parameters are synchronously trained. Two are used to model introns in 5' and 3' UTRs, Three are used to keep track of reading frames across introns. As in SNAP [9] it is ensured that splice sites in coding regions are not predicted in such a way that splicing generates stop codons. This is achieved by special branching in the splice sites and an additional three intron models not shown in Figure 1.

Exons in UTRs are modelled by two sub-models in each UTR. The non-coding part of coding exons and fully non-coding exons are modelled separately. All UTR exon models are homogeneous HMMs with shared third order emissions. N-SCAN and DOUBLESCAN also predict UTRs but use homology information and thus require cross-species alignments for training and decoding. Agene uses the same intron length distribution in UTRs as in coding regions, whereas N-SCAN uses separate distributions. In addition N-SCAN has separate models for first, internal, last, and single exons in UTRs. We have chosen the simpler UTR model because the amount of training material is often limited.

#### Signal modelling

Splice sites are modelled with the HMM equivalent of first order weight array matrices (WAMs) -3 to +8 nucleotides from the donor site and -30 to +3 nucleotides from the acceptor site. This includes the cytosine and thymine rich region (CT-tract) upsteam of the acceptor splice site. For maximal flexibility, no assumptions about splice site consensus are used to prime the weight matrices. The splice sites in UTRs seem to be very similar to the ones in the coding region [28]. For this reason the intron parts of these models share parameters with the splice site models in coding regions. The exon parts of the splice sites are modelled separately. Transcription start, CDS start, and CDS end are modelled as first order weight array matrices of length 7, 14, and 14 respectively. The six bases that constitute the poly-A signal are modelled by a WAM with order increasing from 0 to 5. For a few species including a branch point would strengthen the model. To capture this information, however, a WAM must be primed with a species-specific consensus motif, a step that is not easily automated.

#### Length modelling

Length modelling of gene structure blocks contributes significantly to gene finder performance. HMM gene finders use geometric or explicit duration (ED) modelling [29] or a combination of both. Geometric modelling is computationally cheap but ignores modality in length distributions of gene structure blocks. ED modelling can capture arbitrary length distributions but is computationally expensive. As opposed to geometric modelling where the computation time is proportional to the sequence length *L*, (*O*(*L*)), the computation time of ED modelling is at least proportional to the square of the sequence length (*O*(*L*^2^)). For this reason it is practically infeasible to model long sequences unless the length distributions can be bounded somehow. One solution is to truncate the length distribution not allowing lengths above some reasonable cutoff. Genscan uses ED modelling for coding exons which are naturally bounded by the stop codons in the reading frame. Even with a truncated distribution it is not practically possible to use ED modelling for introns. Many species, however, have a modal distribution of intron lengths, with introns clustering around a certain typical length. Augustus uses ED modelling for a first fixed part of the intron and geometric modelling for the rest. This allows for modality in the distribution of relatively short introns.

**Figure 2:**
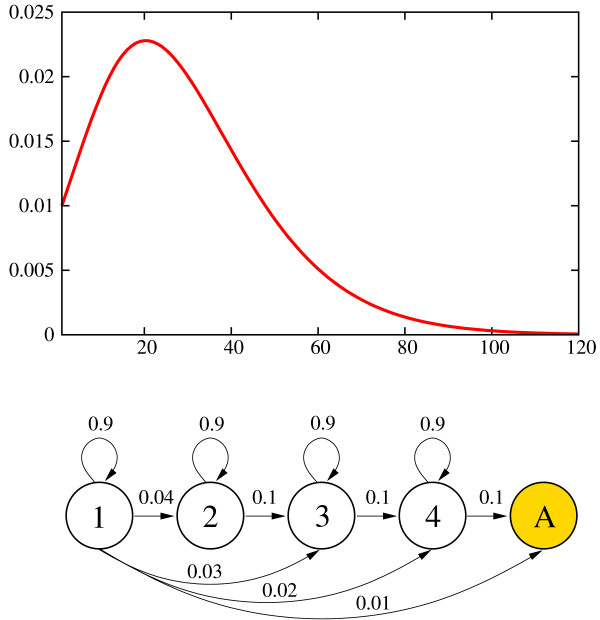
**Example ADPH distribution and probability graph**. ADPH distribution with four phases and associated probability graph. The distribution describes the probability of passing from state one to the absorbing state, A, in a given number of steps. The example constitutes the special case of a mixture of an exponential and three negative binomial distributions. This arises when the loop probabilities are equal.

**Figure 3:**
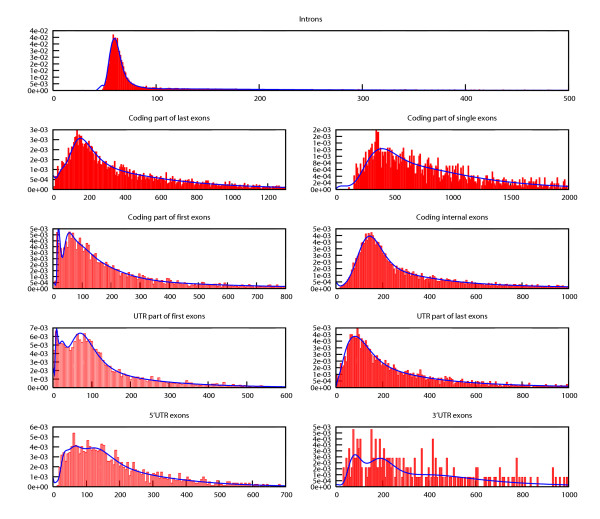
**Example fittings**. Example ADPH fittings to length distribution of *D. melanogaster *gene structure elements. The plots each show actual length distribution in red and ADPH fit in blue.

In contrast to existing gene finders Agene models the full length distribution for all gene structure elements, including introns. The approach we take is to implement this generalised HMM (GHMM) functionality within the standard HMM framework. This is achieved by fitting an HMM state structure to the length distribution of each gene structure element. For this purpose we take advantage of newly developed theory for acyclic discrete phase type (ADPH) distributions [30]. An ADPH distribution describes the probability of moving through a directed acyclic graph with a number of phases (states) in a specified number of steps. For a special subset of sparse graphs there is a one to one correspondence between graph and ADPH distribution. An example of such a graph and its distribution is shown in Figure 2. These graphs conform to the following constraints: Phases are sequentially connected and only the first phase has edges to all other phases. All phases except the last absorbing phase has a loop edge to itself. If the probability associated with the loop edge of phase *i *is denoted *q*_*i *_then the relation *q*_1 _≥ ... ≥ *q*_*i *_≥ ... ≥ *q*_*n *_must apply for all *n *phases. For each variable-sized gene element an ADPH distribution with 15 phases is fitted to the length distribution of the element. The graph underlying the fitted ADPH distribution is then used as HMM state structure for the gene element. The transition probabilities associated with the 15 states (phases) are fixed and not part of the subsequent training. The fitting is done using PhFit [31]. A set of example fittings for *D. melanogaster *genes are shown in Figure 3. 

ED modelling is implemented as drawing from a parametric fitting to a distribution. This means that no matter how many or few parameters the function has, the complexity of the GHMM will be *O*(*L*^2^) because all possible sequence lengths must be considered for each sequence position. When the parametric function is implemented as an ADPH distribution directly in the HMM structure the complexity is reflected by the number of parameters (phases) of the fitted function. Since the number of phases is a constant this allows for GHMM functionality with a linear computational complexity in sequence length (*O*(*L*)).

In theory, an ADPH distribution can accommodate any distribution given enough states. With *L *states it is equivalent to fully-fledged ED modelling (*O*(*L*^2^)). In practice, however, it is not feasible to fit an ADPH with so many phases. ADPHs can give a good fit to a distribution if it ultimately has an exponentially decaying tail and if the modalities in the distribution is located in the first part of the distribution. The non-geometric features modelled, however, are not confined to a fixed interval as in an Augustus type approach. These characteristics are well in line with the nature of sequence length distributions and we have been able to fit all sequence length distributions encountered.

In some training sets there may not be enough examples to fit length distributions for both single, first internal, and last coding exons. There may be too few examples of single coding exons in complex organisms and there may be too few first and last coding exons in simple organisms. In the first case we pool single and internal coding exons in the fitting. In the second case all coding exons are pooled. In cases with few intron examples we use only four phases to model the distribution to avoid over-fitting.

### Generation of training sets

A training set of reliable gene structures is crucial for a machine learning approach to gene finding. It is often unclear to what extent the gene structure annotation submitted to the various databases is based on prediction, inference, or experimental validation. In addition, the degree of curation and confirmation is not always obvious. For these reasons reliable training sets are hard to come by for most organisms. To overcome some of these issues, we generate our own set of gene structure annotations by mapping experimentally obtained mRNAs to the genome. For this study we have used RefSeq mRNA entries with curation label "provisional" or better [32]. The mRNAs are mapped to the genome using BLAT [33]. BLAT is fast and accurate, and makes an effort to pick, among large equivalently scoring gaps, the one conforming to the GT-AG splice-site consensus. In cases where only one possible gap allows alignment of all surrounding mRNA bases the GT-AG consensus is not required. For each mRNA only the best match is considered and this is discarded unless it accounts for 98% of the mRNA sequence allowing for 1% mismatches and 0.5% bases inserted in the mRNA.

Donor and acceptor splice site pairs across introns are analysed to make sure that each pair constitute the only possible set that accounts for the mRNA sequence. This will not be the case if the donor and acceptor site can be shifted in parallel to another G [TC]-AG position producing the same coding sequence. These unambiguous splice sites are found using a tailored Smith-Waterman algorithm similar to EST_GENOME [34] but allowing GC-AG splice sites as well. This flexibility is relevant in at least *C. elegans *[35] and *H. sapiens *[36]. Coding start and end are obtained from CDS annotation of the RefSeq mRNAs. It is checked that start and stop codons conform to the ATG and (TAA, TAG, TGA) consensus and that each annotated CDS in both the mRNA and the gene structure are open reading frames. Finally, the resulting set of gene structures is similarity reduced using the Hobohm 2 algorithm [37]. This reduction is based on WU-BLAST [38] DNA level homologies with an e-value of at most le-03. To contain sufficient information for training, the homology reduced set must contain at least 200 gene structures, and preferably 300 or more (see below).

### Training and decoding

The final model is trained as a Class Hidden Markov model [3]. This allows training of the entire model in one single step by specifying the sequence parts each state is allowed to train on. This has the advantage that transitions between sub-models and non-additive effects of individual sub-models is also trained. 

For many new genomes, not enough full-length mRNAs are available to generate a UTR annotated training set of sufficient size. In these cases the training set is supplemented with gene structures without UTR annotation. This presents a problem in training because unspecific training on un-annotated UTR sequence pollutes the signal contributed by annotated UTRs. To solve this problem two parallel UTR sub-models are introduced (not shown in Figure 1). These parallel sub-models mirror the true UTR models and allows the un-annotated UTRs to be parsed without contributing to the training of the actual UTR sub-models. This way the un-annotated UTR sequence does not interfere with training of UTRs. Transitions from the mirror models to the actual models allow each UTR to contribute to the extent it is annotated. 

Training is done using the standard Baum-Welch algorithm. For decoding we use an N-best algorithm [3]. This finds the most probable prediction summed over all paths yielding the same prediction. This is crucial to our length modelling approach, since the phase type distributions arise as a sum of probabilities of paths returning the same length.

Some genomes such as *H. sapiens *and *M. musculus *are so heterogeneous in GC content that parallel models must be trained on subsets of the training set with different GC content. This procedure is also automated in Agene. The gene model is initially pre-trained on the entire training set. The training set is then split into subsets according to GC content and the pre-trained model is trained again on each subset. Before decoding it is established which GC subset the sequence belongs to and the corresponding model is used for prediction.

## Results

To assess the performance of Agene we have tested it on a diverse set of eukaryotes. For a subset of these species-specific *ab initio *gene finders exist. A thorough evaluation of the existing gene finders is not within the scope of this paper. We have chosen Augustus, Genscan and GeneID and tested these on the species they have been customised for. Genscan, however, was tested on all vertebrates using the Human/vertebrate version. The performance of the most accurate predictor for each species is reported together with the performance of Agene in Table 1. The *H. sapiens *version is trained and decoded on the same GC content subsets as Genscan (0% ≤ 41% ≤ 45% ≤ 53% ≤ 100%). For the *M. musculus *version the subsets 0% ≤ 42% ≤ 46% ≤ 51% ≤ 100% are used. Genscan was run locally with default settings. Augustus and GenelD where run on their respective web servers with "forward strand only" settings. Table 1 also reflects how gene models and use of training material differ for the listed organisms. Each version of Agene was evaluated using six-fold cross validation. To make the evaluation reflect to what extent Agene offers an alternative to non-experts we have not attempted to re-train existing gene finders to the relevant species.

**Figure 4:**
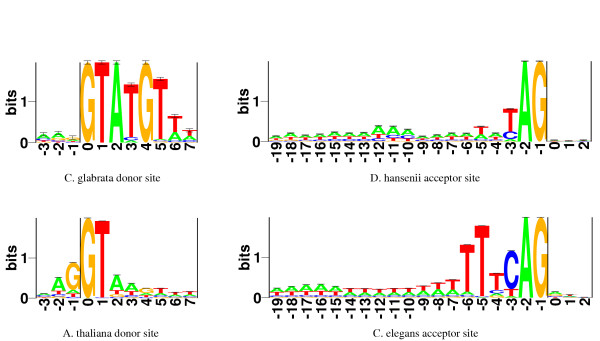
**Logos of donor and acceptor splice sites**. A graphic representation of aligned donor and acceptor splice sites. The relative heights of letters correspond to frequencies of bases at each position. The degree of sequence conservation is reflected in the total height of a stack of letters, measured in bits of information.

The set of species listed in Table 1 represents a diverse selection of gene structures. These vary with respect sequence signals, overall sequence composition, and length of gene elements. The mean GC content of the genes in our training sets ranges from 36% for *C. glabrata *to 53% for *U. maydis*. To illustrate the variation of sequence signals, sequence logos [39] for donor and acceptor splice sites are shown in Figure 4. It is evident that species-specific training of a flexible model is required to accommodate this level of diversity. To further emphasise the importance of species-specificity we have tested the *C. elegans *version of Agene on a few of the other test species. The differences in performance between the *C. elegans *version and the versions created for each species are shown in Table 2.

**Figure 5:**
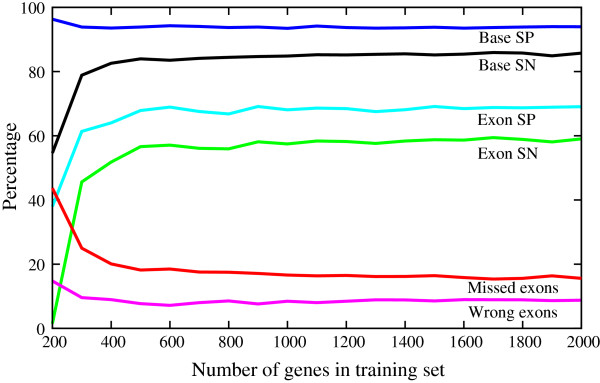
**Performance as a function of training set size**. The plot shows the nucleotide and exon sensitivity and specificity as well as missed and wrong exons as a function of the number of genes in the training set.

The sizes of the training sets used range from 266 for *D. rerio *and 700 for *U. maydis *to 7152 for *N. crassa *and 7914 for *A. nidulans*. The training sets are available as additional files [see Additional file 1, 2, 3, 4, 5, 6, 7, 8, 9, 10, 11, 12, 13, 14, 15, 16, 17, 18, 19, 20, 21, 22, 23, 24, 25, 26, 27, 28]. To investigate how training set size influences performance the *U. maydis *version of Agene was generated using different sized training sets. Performance as a function of training set size is shown in Figure 5. A size of at least 200 gene structures is required to accommodate the large number of parameters in the model. The performance benefits strongly from larger training sets up to a size of about 500 gene structures. Larger training sets only improve performance slightly. These relationships may vary depending on the heterogeneity of gene structures of the species in question. Agene is accessible through our web interface [40].

## Discussion

For the majority of test species the performance of Agene is comparable or slightly better than the alternative gene finders we have tested. We have aimed at developing a method as flexible as possible to accommodate a large variety of genome characteristics and to work with typical sized training sets. This choice, however, does not seem to allow for an effective modelling of gene structures in species with very long introns like *H. sapiens *and *M. musculus*. Our specificity for these species is comparable to that of the remaining test species but the sensitivity is lower. Though important, this category of species does not represent the type of species where automated gene finder generation is most in demand. Species such as *H. sapiens *and *M. musculus *are subject to so much attention that several high quality gene finders already exist. Our focus is on the large number of eukaryotes that do not receive the same attention. The performance of our gene finders are much higher for the genomes they are customised to than for other genomes. These results emphasise the value of a species-specific approach.

The fact that *ab initio *gene prediction methods require a training set limits their use in cases where no mRNA has been sequenced yet. The SNAP gene finder [9] addresses this problem applying a bootstrapping approach that uses a gene finder for a foreign species to create a first prediction. This is then used as virtual training set for the final gene finder. In order to reliably choose a suitable foreign gene finder for bootstrapping extensive information on genome and gene structures is required. This amount of information is often not available for newly sequenced genomes. GeneMark.HMM-ES [5] uses an iterative self-training procedure where the predictions from a previous round serve as training material for the next. Short of species-specific training data these are valid approaches, but bootstrapping methods suffer from the drawback that it is difficult if not impossible to establish what confidence to put in predictions when a reliable and representative test set for the target species is not available.

It has been reported that 40% of *H. sapiens *genes have at least one completely non-coding first exon [41]. Among the other species used in this study UTR exons are common in both *A. thaliana*, *D. malanogaster*, and *M. musculus*. For this reason, Agene returns UTR predictions as an integral part of the gene predictions. This is only possible, however, if the training set used to generate the gene finder contains sufficient UTR annotation. The modelling of the UTR component of the first and last coding exons improves predictions slightly by helping to delineate the start and end of the coding region. We expected that including UTR exons in the model would improve predictions by ensuring that the model does not wrongly predict UTR exons as coding exons. We have tested to what extent this is the case (data not shown) and found the effect on performance is not significant.

The length modelling approach taken in this paper has proved to be an effective way to implement the powers of GHMMs. By integrating length modelling into the HMM state structure computational complexity is linear in the sequence length. This allows for full length modelling of all gene structure elements, including introns.

A problem in evaluating gene predictions stems from the fact that gene finders only predict one full transcript at a time whereas the majority of genes have multiple transcripts. This may account for many of the inconsistencies in predictions. The N-best algorithm, used by Agene, is able to return a number of sub-optimally scoring paths together with the optimal one. It is likely that alternative transcripts are among these sub-optimal predictions. For genes with many alternative exon borders, however, the number of combinations of these is so large that only a subset of these are likely to be be real transcripts. In considering such suboptimal paths a post-processing step is required where the biological sensibility is evaluated.

## Conclusion

A procedure to automatically generate species-specific gene finders for novel genomes is presented. This includes generation of a training set from a set of mRNAs as well as dynamic building and training of an HMM that fits the organism gene structure and the amount annotation available in the training set. Acyclic discrete phase type distributions implemented as HMM state structure are well suited to model sequence length distributions and are very cost effective in terms of complexity. The automatically customised gene finders perform as well or better than most existing manually customised gene finders.

## Supplementary Material

Additional File 1 The sequence part of the training set for *A. gambiae*. The file is in Fasta format and packed using bzip2. Click here for file

Additional File 2 The annotation part of the training set for *A. gambiae*. The file is in GFF format and packed using bzip2. Click here for file

Additional File 3 The sequence part of the training set for *A. nidulans*. The file is in Fasta format and packed using bzip2. Click here for file

Additional File 4 The annotation part of the training set for *A. nidulans*. The file is in GFF format and packed using bzip2. Click here for file

Additional File 5 The sequence part of the training set for *A. thaliana*. The file is in Fasta format and packed using bzip2. Click here for file

Additional File 6 The annotation part of the training set for *A. thaliana*. The file is in GFF format and packed using bzip2. Click here for file

Additional File 7 The sequence part of the training set for *C. elegans*. The file is in Fasta format and packed using bzip2. Click here for file

Additional File 8 The annotation part of the training set for *C. elegans*. The file is in GFF format and packed using bzip2. Click here for file

Additional File 9 The sequence part of the training set for *C. glabrata*. The file is in Fasta format and packed using bzip2. Click here for file

Additional File 10 The annotation part of the training set for *C. glabrata*. The file is in GFF format and packed using bzip2. Click here for file

Additional File 11 The sequence part of the training set for *D. hansenii*. The file is in Fasta format and packed using bzip2. Click here for file

Additional File 12 The annotation part of the training set for *D. hansenii*. The file is in GFF format and packed using bzip2. Click here for file

Additional File 13 The sequence part of the training set for *D. melanogaster*. The file is in Fasta format and packed using bzip2. Click here for file

Additional File 14 The annotation part of the training set for *D. melanogaster*. The file is in GFF format and packed using bzip2. Click here for file

Additional File 15 The sequence part of the training set for *D. rerio*. The file is in Fasta format and packed using bzip2. Click here for file

Additional File 16 The annotation part of the training set for *D. rerio*. The file is in GFF format and packed using bzip2. Click here for file

Additional File 17 The annotation part of the training set for *H. sapiens*. The file is in GFF format and packed using bzip2. Click here for file

Additional File 18 The sequence part (sub-file 1) of the training set for *H. sapiens*. The file is in Fasta format and packed using bzip2. Click here for file

Additional File 19 The sequence part (sub-file 2) of the training set for *H. sapiens*. The file is in Fasta format and packed using bzip2. Click here for file

Additional File 20 The annotation part (sub-file 3) of the training set for *H. sapiens*. The file is in Fasta format and packed using bz2. Click here for file

Additional File 21 The sequence part of the training set for *K. lactis*. The file is in Fasta format and packed using bzip2. Click here for file

Additional File 22 The annotation part of the training set for *K. lactis*. The file is in GFF format and packed using bzip2. Click here for file

Additional File 23 The sequence part of the training set for *M. musculus*. The file is in Fasta format and packed using bzip2. Click here for file

Additional File 24 The annotation part of the training set for *M. musculus*. The file is in GFF format and packed using bzip2. Click here for file

Additional File 25 The sequence part of the training set for *N. crassa*. The file is in Fasta format and packed using bzip2. Click here for file

Additional File 26 The annotation part of the training set for *N. crassa*. The file is in GFF format and packed using bzip2. Click here for file

Additional File 27 The sequence part of the training set for *U. maydis*. The file is in Fasta format and packed using bzip2. Click here for file

Additional File 28 The annotation part of the training set for *U. maydis*. The file is in GFF format and packed using bzip2. Click here for file

## Figures and Tables

**Table 1:** Performance evaluation. NSN: nucleotide sensitivity, NSP: nucleotide specificity, ESN: exon sensitivity, ESP: exon specificity, ME: missed exons, WE: wrong exons. NSN is defined as the percentage of annotated coding bases predicted as coding and NSP as the percentage of predicted coding bases annotated as as coding. ESN and ESP reflect analogously how well the methods predict exons exactly right. Superscripts on the species names indicate the type of generated gene model. 1: Full model. 2: 3' UTR exons and introns not modelled. 3: No UTR exons and introns modelled. 4: UTR exons and introns as well as UTR part of first and last exons not modelled. Subscripts indicate whether shared length distributions are used. 1: No shared distributions. 2: Internal and single coding exons share distribution. 3: all coding coding exons share distribution

Species	Predictor	NSN	NSP	ESN	ESP	ME	WE
C. elegans23 MathType@MTEF@5@5@+=feaafiart1ev1aaatCvAUfKttLearuWrP9MDH5MBPbIqV92AaeXatLxBI9gBaebbnrfifHhDYfgasaacH8akY=wiFfYdH8Gipec8Eeeu0xXdbba9frFj0=OqFfea0dXdd9vqai=hGuQ8kuc9pgc9s8qqaq=dirpe0xb9q8qiLsFr0=vr0=vr0dc8meaabaqaciaacaGaaeqabaqabeGadaaakeaacqWGdbWqcqGGUaGlcaaMc8UaemyzauMaemiBaWMaemyzauMaem4zaCMaemyyaeMaemOBa4Maem4Cam3aa0baaSqaaiabikdaYaqaaiabiodaZaaaaaa@3BBA@	Agene	**95**	**92**	**86**	**85**	**5**	**9**
	GeneID	95	86	75	68	6	16
D. melanogaster11 MathType@MTEF@5@5@+=feaafiart1ev1aaatCvAUfKttLearuWrP9MDH5MBPbIqV92AaeXatLxBI9gBaebbnrfifHhDYfgasaacH8akY=wiFfYdH8Gipec8Eeeu0xXdbba9frFj0=OqFfea0dXdd9vqai=hGuQ8kuc9pgc9s8qqaq=dirpe0xb9q8qiLsFr0=vr0=vr0dc8meaabaqaciaacaGaaeqabaqabeGadaaakeaacqWGebarcqGGUaGlcaaMc8UaemyBa0MaemyzauMaemiBaWMaemyyaeMaemOBa4Maem4Ba8Maem4zaCMaemyyaeMaem4CamNaemiDaqNaemyzauMaemOCai3aa0baaSqaaiabigdaXaqaaiabigdaXaaaaaa@42A9@	Agene	**87**	**87**	**64**	**68**	**13**	**15**
	Augustus	85	91	67	72	15	11
A. thaliana11 MathType@MTEF@5@5@+=feaafiart1ev1aaatCvAUfKttLearuWrP9MDH5MBPbIqV92AaeXatLxBI9gBaebbnrfifHhDYfgasaacH8akY=wiFfYdH8Gipec8Eeeu0xXdbba9frFj0=OqFfea0dXdd9vqai=hGuQ8kuc9pgc9s8qqaq=dirpe0xb9q8qiLsFr0=vr0=vr0dc8meaabaqaciaacaGaaeqabaqabeGadaaakeaacqWGbbqqcqGGUaGlcaaMc8UaemiDaqNaemiAaGMaemyyaeMaemiBaWMaemyAaKMaemyyaeMaemOBa4Maemyyae2aa0baaSqaaiabigdaXaqaaiabigdaXaaaaaa@3CFF@	Agene	**85**	**89**	**67**	**74**	**14**	**11**
	Genscan	82	83	49	51	17	18
N. crassa14 MathType@MTEF@5@5@+=feaafiart1ev1aaatCvAUfKttLearuWrP9MDH5MBPbIqV92AaeXatLxBI9gBaebbnrfifHhDYfgasaacH8akY=wiFfYdH8Gipec8Eeeu0xXdbba9frFj0=OqFfea0dXdd9vqai=hGuQ8kuc9pgc9s8qqaq=dirpe0xb9q8qiLsFr0=vr0=vr0dc8meaabaqaciaacaGaaeqabaqabeGadaaakeaacqWGobGtcqGGUaGlcaaMc8Uaem4yamMaemOCaiNaemyyaeMaem4CamNaem4CamNaemyyae2aa0baaSqaaiabigdaXaqaaiabisda0aaaaaa@3A83@	Agene	**76**	**95**	**57**	**76**	**25**	**7**
	GeneID	24	95	14	83	76	8
A. nidulans24 MathType@MTEF@5@5@+=feaafiart1ev1aaatCvAUfKttLearuWrP9MDH5MBPbIqV92AaeXatLxBI9gBaebbnrfifHhDYfgasaacH8akY=wiFfYdH8Gipec8Eeeu0xXdbba9frFj0=OqFfea0dXdd9vqai=hGuQ8kuc9pgc9s8qqaq=dirpe0xb9q8qiLsFr0=vr0=vr0dc8meaabaqaciaacaGaaeqabaqabeGadaaakeaacqWGbbqqcqGGUaGlcaaMc8UaemOBa4MaemyAaKMaemizaqMaemyDauNaemiBaWMaemyyaeMaemOBa4Maem4Cam3aa0baaSqaaiabikdaYaqaaiabisda0aaaaaa@3D3F@	Agene	**84**	**93**	**59**	**70**	**19**	**9**
	GeneID	88	86	49	50	18	19
H. sapiens11 MathType@MTEF@5@5@+=feaafiart1ev1aaatCvAUfKttLearuWrP9MDH5MBPbIqV92AaeXatLxBI9gBaebbnrfifHhDYfgasaacH8akY=wiFfYdH8Gipec8Eeeu0xXdbba9frFj0=OqFfea0dXdd9vqai=hGuQ8kuc9pgc9s8qqaq=dirpe0xb9q8qiLsFr0=vr0=vr0dc8meaabaqaciaacaGaaeqabaqabeGadaaakeaacqWGibascqGGUaGlcaaMc8Uaem4CamNaemyyaeMaemiCaaNaemyAaKMaemyzauMaemOBa4Maem4Cam3aa0baaSqaaiabigdaXaqaaiabigdaXaaaaaa@3BE6@	Agene	**63**	**56**	**47**	**40**	**38**	**48**
	Genscan	87	60	63	47	14	39
M. musculus12 MathType@MTEF@5@5@+=feaafiart1ev1aaatCvAUfKttLearuWrP9MDH5MBPbIqV92AaeXatLxBI9gBaebbnrfifHhDYfgasaacH8akY=wiFfYdH8Gipec8Eeeu0xXdbba9frFj0=OqFfea0dXdd9vqai=hGuQ8kuc9pgc9s8qqaq=dirpe0xb9q8qiLsFr0=vr0=vr0dc8meaabaqaciaacaGaaeqabaqabeGadaaakeaacqWGnbqtcqGGUaGlcaaMc8UaemyBa0MaemyDauNaem4CamNaem4yamMaemyDauNaemiBaWMaemyDauNaem4Cam3aa0baaSqaaiabigdaXaqaaiabikdaYaaaaaa@3D97@	Agene	**73**	**88**	**56**	**68**	**28**	**14**
	Genscan	88	82	69	68	14	17
D. rerio23 MathType@MTEF@5@5@+=feaafiart1ev1aaatCvAUfKttLearuWrP9MDH5MBPbIqV92AaeXatLxBI9gBaebbnrfifHhDYfgasaacH8akY=wiFfYdH8Gipec8Eeeu0xXdbba9frFj0=OqFfea0dXdd9vqai=hGuQ8kuc9pgc9s8qqaq=dirpe0xb9q8qiLsFr0=vr0=vr0dc8meaabaqaciaacaGaaeqabaqabeGadaaakeaacqWGebarcqGGUaGlcaaMc8UaemOCaiNaemyzauMaemOCaiNaemyAaKMaem4Ba82aa0baaSqaaiabikdaYaqaaiabiodaZaaaaaa@392E@	Agene	**88**	**89**	**67**	**70**	**13**	**13**
	Genscan	91	87	67	69	12	10
A. gambiae13 MathType@MTEF@5@5@+=feaafiart1ev1aaatCvAUfKttLearuWrP9MDH5MBPbIqV92AaeXatLxBI9gBaebbnrfifHhDYfgasaacH8akY=wiFfYdH8Gipec8Eeeu0xXdbba9frFj0=OqFfea0dXdd9vqai=hGuQ8kuc9pgc9s8qqaq=dirpe0xb9q8qiLsFr0=vr0=vr0dc8meaabaqaciaacaGaaeqabaqabeGadaaakeaacqWGbbqqcqGGUaGlcaaMc8Uaem4zaCMaemyyaeMaemyBa0MaemOyaiMaemyAaKMaemyyaeMaemyzau2aa0baaSqaaiabigdaXaqaaiabiodaZaaaaaa@3B82@	Agene	**81**	**83**	**52**	**63**	**18**	**15**
D. hansenii24 MathType@MTEF@5@5@+=feaafiart1ev1aaatCvAUfKttLearuWrP9MDH5MBPbIqV92AaeXatLxBI9gBaebbnrfifHhDYfgasaacH8akY=wiFfYdH8Gipec8Eeeu0xXdbba9frFj0=OqFfea0dXdd9vqai=hGuQ8kuc9pgc9s8qqaq=dirpe0xb9q8qiLsFr0=vr0=vr0dc8meaabaqaciaacaGaaeqabaqabeGadaaakeaacqWGebarcqGGUaGlcaaMc8UaemiAaGMaemyyaeMaemOBa4Maem4CamNaemyzauMaemOBa4MaemyAaKMaemyAaK2aa0baaSqaaiabikdaYaqaaiabisda0aaaaaa@3D27@	Agene	**82**	**85**	**64**	**68**	**17**	**19**
K. lactis34 MathType@MTEF@5@5@+=feaafiart1ev1aaatCvAUfKttLearuWrP9MDH5MBPbIqV92AaeXatLxBI9gBaebbnrfifHhDYfgasaacH8akY=wiFfYdH8Gipec8Eeeu0xXdbba9frFj0=OqFfea0dXdd9vqai=hGuQ8kuc9pgc9s8qqaq=dirpe0xb9q8qiLsFr0=vr0=vr0dc8meaabaqaciaacaGaaeqabaqabeGadaaakeaacqWGlbWscqGGUaGlcaaMc8UaemiBaWMaemyyaeMaem4yamMaemiDaqNaemyAaKMaem4Cam3aa0baaSqaaiabiodaZaqaaiabisda0aaaaaa@3A87@	Agene	**78**	**91**	**69**	**79**	**21**	**11**
C. glabrata34 MathType@MTEF@5@5@+=feaafiart1ev1aaatCvAUfKttLearuWrP9MDH5MBPbIqV92AaeXatLxBI9gBaebbnrfifHhDYfgasaacH8akY=wiFfYdH8Gipec8Eeeu0xXdbba9frFj0=OqFfea0dXdd9vqai=hGuQ8kuc9pgc9s8qqaq=dirpe0xb9q8qiLsFr0=vr0=vr0dc8meaabaqaciaacaGaaeqabaqabeGadaaakeaacqWGdbWqcqGGUaGlcaaMc8Uaem4zaCMaemiBaWMaemyyaeMaemOyaiMaemOCaiNaemyyaeMaemiDaqNaemyyae2aa0baaSqaaiabiodaZaqaaiabisda0aaaaaa@3D05@	Agene	**91**	**94**	**82**	**82**	**9**	**9**
U. maydis14 MathType@MTEF@5@5@+=feaafiart1ev1aaatCvAUfKttLearuWrP9MDH5MBPbIqV92AaeXatLxBI9gBaebbnrfifHhDYfgasaacH8akY=wiFfYdH8Gipec8Eeeu0xXdbba9frFj0=OqFfea0dXdd9vqai=hGuQ8kuc9pgc9s8qqaq=dirpe0xb9q8qiLsFr0=vr0=vr0dc8meaabaqaciaacaGaaeqabaqabeGadaaakeaacqWGvbqvcqGGUaGlcaaMc8UaemyBa0MaemyyaeMaemyEaKNaemizaqMaemyAaKMaem4Cam3aa0baaSqaaiabigdaXaqaaiabisda0aaaaaa@3AA5@	Agene	**87**	**94**	**61**	**69**	**14**	**9**

**Table 2:** Cross-species performance. Performance of Agene for *C. elegans *on a selection of other test species. The percentages shown are the differences in performance relative to the versions of Agene that are generated for the species on question. NSN: nucleotide sensitivity, NSP: nucleotide specificity, ESN: exon sensitivity, ESP: exon specificity, ME: missed exons, WE: wrong exons

Species	NSN	NSP	ESN	ESP	ME	WE
*A. thaliana*	-21	0	-29	-10	22	1
*D. melanogaster*	-8	-5	-21	-17	8	8
*N. crassa*	-7	-19	-34	-45	5	25
*D. hansenii*	-26	-3	-52	-27	16	6
*D. rerio*	-33	-2	-33	-8	31	1
*C. glabrata*	-21	-7	-58	-38	13	13
